# Is there a role for immune checkpoint inhibitors in high-risk medulloblastoma beyond the salvage setting?

**DOI:** 10.1093/oncolo/oyag386

**Published:** 2026-10-01

**Authors:** Yi-Lun Wang, Chieh-Tsai Wu, Wei-Heng Kao, Tsung-Yen Chang, Tang-Her Jaing

**Affiliations:** Division of Hematology/Oncology, Department of Pediatrics, Chang Gung Memorial Hospital, Chang Gung University, Linkou, Taoyuan, 33305, Taiwan; Department of Surgery, Division of Neurosurgery, Chang Gung Memorial Hospital, Chang Gung University, Linkou, Taoyuan, 33305, Taiwan; Department of Radiation Oncology, Chang Gung Memorial Hospital, Linkou, Taoyuan, 33305, Taiwan; Division of Hematology/Oncology, Department of Pediatrics, Chang Gung Memorial Hospital, Chang Gung University, Linkou, Taoyuan, 33305, Taiwan; Division of Hematology/Oncology, Department of Pediatrics, Chang Gung Memorial Hospital, Chang Gung University, Linkou, Taoyuan, 33305, Taiwan

**Keywords:** Medulloblastoma, Immune Checkpoint Inhibitors, Immunotherapy, Drug-Related Side Effects and Adverse Reactions, Pediatrics

We read with interest the systematic review by Fontaão and colleagues, which synthesizes immunotherapy outcomes across 56 studies and at least 183 patients with medulloblastoma.^1^ Every included patient had recurrent, refractory, relapsed, high-risk, or disseminated disease, and immunotherapy was almost uniformly administered as salvage. We highlight a distinct and increasingly common setting that their search could not capture: immune checkpoint inhibitor (ICI) maintenance after upfront high-dose chemotherapy and tandem autologous hematopoietic cell transplantation (HCT) in high-risk medulloblastoma.^2^ This post-transplant, irradiated host differs immunologically and toxicologically from the salvage populations the review summarizes.

Our group treated three children with high-risk medulloblastoma with sequential pembrolizumab and nivolumab maintenance after tandem autologous HCT (Table 1). Grade 3 or higher immune-related pneumonitis developed in two of the three patients, forcing permanent discontinuation of therapy; immune-mediated thrombocytopenia and colitis were also observed. No disease benefit was attributable to immunotherapy. All events resolved or stabilized with ICI discontinuation and immunosuppression, without treatment-related death. This granular, setting-specific toxicity reporting is precisely what Fontaão et al. found lacking, as only 11 of the included studies reported adverse events specific to medulloblastoma.^1^ Our observations suggest the risk side of the ICI equation may be heavier in the transplanted, irradiated host than the salvage-setting literature implies.^3^

**Table 1.** Clinical characteristics and CTCAE-aligned safety profile of pediatric patients with medulloblastoma receiving immune checkpoint inhibitor maintenance after tandem autologous hematopoietic cell transplantation.

Case	Age, y/sex	Surgery	Molecular subtype	**Radiotherapy, GyE (fractions)** ^a^	Chemotherapy and tandem HDT–ASCT	**Checkpoint inhibitor (cycles)** ^b^	Indication for ICI	irAE	**CTCAE grade** ^c^	Management	Outcome
**1**	10.7/F	STR	Non-WNT/non-SHH	Tumor bed 54 (30); CSI 23.4 (13)	VCEC/VICE × 6, then HDT–ASCT × 2 (CTE/CyMel)	Pembrolizumab (15) → nivolumab (4)	Residual disease; nivolumab started after pembrolizumab-associated immune thrombocytopenia	Thrombocytopenia	4	ICI discontinuation; methylprednisolone	Resolved
								Pneumonitis	2	FAM regimen	Stable
**2**	12.6/M	NTR	SHH, *TP53*-mutant	Tumor bed 54 (30); CSI 36 (20)	VCEC/VICE × 6, then HDT–ASCT × 2 (CTE/CyMel)	Pembrolizumab (11) → nivolumab (10)		Colitis	2	Medical management; ICI discontinued	Resolved
								Pneumonitis	3	FAM regimen; ICI discontinued	Recovered; oxygen independent
**3**	13.5/M	NTR	Non-WNT/non-SHH	Tumor bed 54 (30); CSI 36 (20)	VCEC/VICE × 6, then HDT–ASCT × 2 (CTE/CyMel)	Pembrolizumab (9)		Pneumonitis	3	FAM regimen; ICI discontinued	Improved; oxygen independent

Abbreviations: ASCT, autologous stem cell transplantation; CSI, craniospinal irradiation; CTCAE, Common Terminology Criteria for Adverse Events; CTE and CyMel, sequential high-dose conditioning regimens used for the two transplants; F, female; FAM, fluticasone, azithromycin, and montelukast; GyE, gray equivalent; HDT, high-dose therapy; ICI, immune checkpoint inhibitor; irAE, immune-related adverse event; M, male; NTR, near-total resection; SHH, sonic hedgehog; STR, subtotal resection; *TP53*, tumor protein p53 gene; VCEC and VICE, induction chemotherapy regimens; WNT, wingless-type; y, year.

a Radiotherapy is reported as total dose in gray equivalent (GyE) with the number of fractions in parentheses.

b Numbers in parentheses after each agent indicate the number of cycles administered.

c Adverse events were graded according to the Common Terminology Criteria for Adverse Events (CTCAE), version 5.0.

These cases also illustrate the biomarker-agnostic prescribing the authors rightly criticize. Two patients had non-WNT/non-SHH disease, and one had SHH-activated, TP53-mutant disease; PD-L1 expression, where assessed, was low. Medulloblastoma is generally an immunologically cold tumor with a low mutational burden,^4^ and such tumors respond poorly to single-agent checkpoint blockade without combination strategies to convert them to a hot phenotype.^5^ Checkpoint blockade in pediatric high-grade CNS malignancies has yielded predominantly progressive disease,^6^ while durable responses have clustered almost exclusively in biologically distinct, hypermutated contexts such as constitutional mismatch repair deficiency.^7^ Exposing heavily pretreated, transplanted children to checkpoint blockade without a defensible biomarker rationale risks meaningful toxicity for improbable benefit, reinforcing the authors’ call to restrict these agents to molecularly selected subsets.

Building on the review’s priorities, we suggest that maintenance and consolidation strategies be studied as a distinct category from salvage therapy, with prospective, tumor- and setting-specific safety capture, pneumonitis foremost, and attention to interactions between prior craniospinal irradiation and checkpoint blockade.^8^ Radiotherapy can prime anti-tumor T-cell responses and help convert cold tumors to a hot phenotype,^9^ and combined with checkpoint blockade may help overcome resistance to checkpoint inhibition,^10^ though optimal dose, fractionation, and sequencing remain undefined and must be weighed against the neurocognitive and endocrine cost of craniospinal irradiation in children. Long-term neurocognitive and endocrine follow-up should be embedded in future trials, given the well-documented vulnerabilities of medulloblastoma survivors.^2^ With these additions, the biomarker-driven framework the authors propose can be extended to the post-transplant setting, where enthusiasm for immunotherapy currently outpaces the evidence supporting it.

We congratulate the authors on a valuable and clarifying contribution.
